# Ferritin nanoparticle vaccine displaying optimized spike protein confers broad protection against Omicron subvariants

**DOI:** 10.3389/fcimb.2025.1676592

**Published:** 2025-11-04

**Authors:** Sheng Feng, Xiao-Yang Yu, Hai-Tong Wang, Zhuo-Xin Li, Shan-Zhi Li, Hui-Yan Wang, Zhuo Ha, Wei Wang

**Affiliations:** ^1^ Jilin Collaborative Innovation Center for Antibody Engineering, Jilin Medical University, Jilin, China; ^2^ Changchun Veterinary Research Institute, Chinese Academy of Agricultural Sciences, Changchun, China; ^3^ Institute of Virology, Wenzhou University, Wenzhou, China

**Keywords:** immune-evasive, ferritin nanoparticles, immunogenicity, cross-neutralizing, Omicron

## Abstract

**Introduction:**

The newly emerged Omicron subvariants demonstrate resistance to current therapeutic antibodies and an enhanced ability to evade the vaccine-induced immune responses. Among them, JN.1 sublineages are considered highly immune-evasive, underscoring the urgent need for broadly protective vaccines. Ferritin nanoparticles, with their unique hollow nanocage structure, provide an efficient antigen-display platform for next-generation vaccine development.

**Methods:**

Based on the previously constructed Delta-6P-S recombinant protein vaccine with broad-spectrum protective effects, this study optimized the S protein structure displayed on the surface of ferritin nanoparticles by comparing the immune responses induced in C57BL/6J mice.

**Results:**

Delta-4S1158 nanoparticles, containing a truncated S-6P structure with four additional mutation sites, elicited robust S-specific immunoglobulin G (IgG), potent neutralizing antibodies, and a Th2-biased T-cell response in C57BL/6J mice, demonstrating favorable immunogenicity and safety. The JN.1-4S1158 nanoparticles, based on this structural design, induced a strong cross-neutralizing antibody response in C57BL/6J mice and conferred effective protection against Omicron BA.5, XBB, and JN.1 variants. Vaccinated mice exhibited significantly reduced viral genomic loads in trachea and lung tissues compared to controls, with no infectious virus detected. Lung tissue pathology was minimal in vaccinated mice.

**Conclusion:**

The JN.1-4S1158 nanoparticle vaccine demonstrates broad-spectrum protective effects against Omicron subvariants and shows potential for further development. It also provides a basis for the development of a universal SARS-CoV-2 vaccine.

## 1 Introduction

Although the emergency phase of the COVID-19 pandemic has been declared over by the World Health Organization (Wang et al., 2024), SARS-CoV-2 continues to evolve and spread (Wang and Guo, 2023). Since the emergence of the Omicron variant, it has spread rapidly worldwide, giving rise to numerous subvariants that have caused widespread infection (Fahrbach et al., 2025; Wasim et al., 2025). Vaccination has long been an effective strategy to combat infectious disease outbreaks (Mambelli and de Araujo, 2025; Moore et al., 2025). Although various vaccine platforms have achieved different degrees of immune protection, they also exhibit inherent limitations (Pischel et al., 2024; Widyaningsih and Hakim, 2024).

Advances in SARS-CoV-2 structural biology and gene delivery technologies offer new opportunities to develop vaccines capable of conferring broader protection against diverse variants (Chen and Farzan, 2025; Holmes, 2024). Recent studies increasingly demonstrate that nanoparticle-mediated antigen delivery systems can significantly enhance immune responses compared with monomeric antigens (Chen et al., 2025; Sun et al., 2025; Wu et al., 2025). Additionally, nanoparticle vaccines have a favorable safety profile and can stimulate robust humoral and cellular immune responses, particularly strong Th2-biased immunity (Belghith et al., 2025; Chiba et al., 2024; Lin et al., 2025). Among these platforms, ferritin-based nanoparticles show particular promise as vaccine carriers (Cao et al., 2024; Chen et al., 2024). Ferritin is a naturally occurring spherical nanocage composed of 24 symmetrically arranged subunits with a conserved structure across species (Ahmadivand and Fux, 2024; Lucignano and Ferraro, 2024). Studies have shown that ferritin nanoparticle vaccines induce T follicular helper cell responses that are three to four times higher than those of traditional subunit vaccines, while maintaining an excellent safety profile (Wang et al., 2023; Widge and Hofstetter, 2023). Therefore, ferritin nanoparticle vaccines represent a promising design strategy with substantial research potential (Yang et al., 2024).

We previously reported a candidate vaccine, Delta-6P-S, with broad-spectrum protective effects (Feng et al., 2023). This immunogen incorporates a T4 fibrin trimerization tag and six proline substitutions, stabilizing the spike protein in its pre-fusion conformation. It exhibited excellent immunogenicity and cross-neutralization activity against wild-type, Beta, Delta, and Omicron variants in C57BL/6J mice and golden hamsters. It protected the hamsters against Delta and Omicron challenges. In the present study, we designed structural variants based on this immunogen, displayed them on the surface of ferritin nanoparticles, and evaluated the induced immune response in C57BL/6J mice. After identifying the spike protein structure most suitable for ferritin display, we developed a nanoparticle vaccine, “JN.1-4S1158”, based on the Omicron JN.1 spike sequence. This vaccine demonstrated strong immunogenicity and cross-neutralization activity against Omicron BA.5, XBB, and JN.1 variants in C57BL/6J mice and conferred protection against challenge with these variants.

## 2 Materials and methods

### 2.1 Cell culture

CHO-S cells were purchased from Thermo Fisher Scientific and cultured in ExpiCHO Expression Medium (Thermo Fisher Scientific, USA). Vero-E6 cells were cryopreserved in our laboratory and cultured in Dulbecco’s modified Eagle’s medium (DMEM) (Thermo Fisher Scientific, USA) supplemented with 10% heat-inactivated fetal bovine serum (FBS) (Thermo Fisher Scientific, USA) and 1% penicillin–streptomycin. The cells were maintained in a 37°C incubator.

### 2.2 Animals and viruses

Specific pathogen-free (SPF) 6-week-old female C57BL/6J mice (18–20 g) were obtained from Beijing Charles River Laboratory Animal Technology Co., Ltd. The authentic Delta (CSTR.16698.06.NPRC6.CCPM-B-V-049-2105-6), BA.5 (SARS-CoV-2 strain Omicron CoV/human/CHN_CVRI-12/2022), XBB (SARS-CoV-2 strain Omicron CoV/human/CHN_CVRI-10/2023), and JN.1 (SARS-CoV-2 strain Omicron CoV/human/CHN_CVRI-05/2024) strains were isolated from patients with COVID-19. All virus experiments were conducted in a Biosafety Level 3 laboratory with standard operating procedures.

### 2.3 Design and preparation of ferritin nanoparticles

The *Helicobacter pylori* ferritin sequence was obtained from the National Center for Biotechnology Information (https://www.ncbi.nlm.nih.gov/). The spike protein sequences of Delta and Omicron JN.1 variants were designed according to mutation sites reported by the World Health Organization (https://www.who.int/). Six proline substitutions were introduced at residues 817, 892, 899, 942, 986, and 987. Four additional mutations (P1143S, F1148I, Y1155I, and F1156H) were also introduced. The furin cleavage site (682-RRAR-686) was replaced with 682-GSAS-686. The construct also contained an N-terminal Kozak sequence and signal peptide, and a C-terminal T4 fibritin trimerization tag fused to *H. pylori* ferritin. Ferritin nanoparticles were expressed in CHO-S cells cultured in ExpiCHO Expression Medium at 37°C with agitation at 125 rpm in a humidified atmosphere containing 8% CO_2_. Nanoparticles were purified by affinity chromatography using Strep-Tactin^®^XT (IBA Life Sciences, Germany) with a gravity-flow column. The target proteins were eluted with Strep-Tactin^®^XT Elution Buffer (IBA Life Sciences, Germany) and resuspended in phosphate-buffered saline (PBS) for storage at −80°C.

### 2.4 Western blot

Ferritin nanoparticle samples were mixed with an appropriate amount of 5× loading buffer, heated at 100°C for 10min, and separated on a 7.5% polyacrylamide Tris-glycine gel for 2h at 80V. Then, the immunoblotting was transferred to the NC membrane for 90min at 100V. The membrane was sealed overnight at 4°C in TBS and 0.1% Tween 20 (TBST) with 5% bovine serum albumin (BSA) and then incubated at room temperature with a 1:3,000 dilution of the RBD pAb and strep-tag mAb (Thermo Fisher Scientific, USA) for 2.5h. After four washes with TBST, the membranes were incubated for 1h at room temperature with the horseradish peroxidase (HRP)-labeled goat anti-mouse immunoglobulin G (IgG) (Beyotime, China, 1:5,000 dilution). The membranes were developed with SuperSignal West Pico Chemiluminescent Substrate (Thermo Fisher Scientific, USA), and images were acquired with Amersham Imager 600 (General Electric Company, USA).

### 2.5 Immunization of C57BL/6J mice

To compare immune responses induced by different nanoparticle designs, 25 female C57BL/6J mice were divided into five groups (*n* = 5). Mice were immunized with three doses of Delta-6P-S, Delta-S1208, Delta-S1158, or Delta-4S1158 nanoparticle vaccines at weeks 3, 6, and 9. Control groups received alum alone. Tail-vein blood was collected every 3 weeks, and spleens were harvested 3 weeks after the final immunization.

To evaluate the immunogenicity of JN.1-4S1158, 30 female C57BL/6J mice were divided into two groups (*n* = 15). Mice in the immunized group received three doses of JN.1-4S1158 nanoparticles at weeks 3, 6, and 9, while controls received alum. Tail-vein blood was collected every 3 weeks.

### 2.6 Omicron variant challenge in C57BL/6J mice

Three groups of JN.1-4S1158-immunized and three groups of alum-treated C57BL/6J mice (*n*=5 per group) were anesthetized with 3% isoflurane inhalation and inoculated intranasally with 100 μL of 10^4.75^ TCID_50_/mL Omicron BA.5, XBB, and JN.1 variants. Mice were euthanized by cervical dislocation 4 days post-challenge (dpi). Lungs and trachea were harvested for measurement of viral titers and gene copies at 4 dpi. Lung pathology was compared between vaccinated and control groups.

### 2.7 ELISA

The S-specific IgG antibody ELISA (enzyme-linked immunosorbent assay) was performed as described earlier. Plates coated with spike proteins were used (Sangon Biotech, China). Serial dilutions of serum were added for incubation for 50min at 37°C. After washing, HRP‐conjugated goat anti-mouse IgG antibodies (ZSGB-BIO, China) diluted 1:10,000 with PBS were added and incubated for 30min at 37°C. TMB Single-Component Substrate solution was used for color development. The reaction was terminated using 2% H_2_SO_4_, and the optical density (OD) was measured at 450 nm.

### 2.8 Neutralization of Omicron live virus and determination of TCID_50_

Neutralization assays were performed with Omicron BA.5, XBB, and JN.1 variants. Diluted sera were incubated with 100 TCID_50_ of live Omicron virus at 37°C for 1h, then added to Vero-E6 cells (5 × 10^3^/well). Cultures were incubated for 4 days, and cytopathic effects (CPEs) were assessed microscopically. Neutralization titers were reported as the highest serum dilution achieving 50% neutralization.

For TCID_50_ determination, Vero-E6 cells (5 × 10³/well) were seeded in 96-well plates overnight. Serially diluted tissue homogenates (100 µL/well) were added and incubated for 4 days. CPE was recorded using a microscope.

### 2.9 Quantitative real-time PCR

The HiScript II U+ One Step qRT-PCR (quantitative real-time polymerase chain reaction) Probe Kit (Vazyme, China) was used for qPCR of viral RNA detection. Primers and probes targeting the N gene and sgE gene of SARS-CoV-2 were synthesized by Sangon Biotech Co., Ltd. (Shanghai, China). Viral gene loads were quantified by TaqMan qRT-PCR as previously described (Li et al., 2022).

### 2.10 Flow cytometry

To analyze T-cell response (CD3^+^CD4^+^ and CD3^+^CD8^+^) and cytokine production, 2 × 10^6^ splenic lymphocytes from each mouse were stimulated in 24-well plates with 10 μg/well SARS-CoV-2 spike peptide pools (Sino Biological, China) for 12h. Cells were stained on ice with surface antibodies, including PE/Cyanine7 anti-mouse CD3ϵ Antibody (BioLegend, USA), FITC anti-mouse CD4 Antibody (BioLegend, USA), and APC anti-mouse CD8a Antibody (BioLegend, USA). After washing, cells were fixed and permeabilized (BD, USA) for 30min, washed in 1×Perm/Wash™ buffer, and stained intracellularly with PE anti-mouse IFN-γ Antibody (BioLegend, USA) and PE anti-mouse IL-4 Antibody (BioLegend, USA). Data were acquired on a Beckman flow cytometer.

### 2.11 Statistical analysis

GraphPad Prism v.8.0.2 was used for all statistical analyses, and intergroup differences were compared by multiple *t*-tests. Antibody titer data were log-transformed before analysis. Correlations were assessed by Spearman rank-correlation tests.

## 3 Results

### 3.1 Design, expression, and purification of ferritin nanoparticles

**Figure 1:**
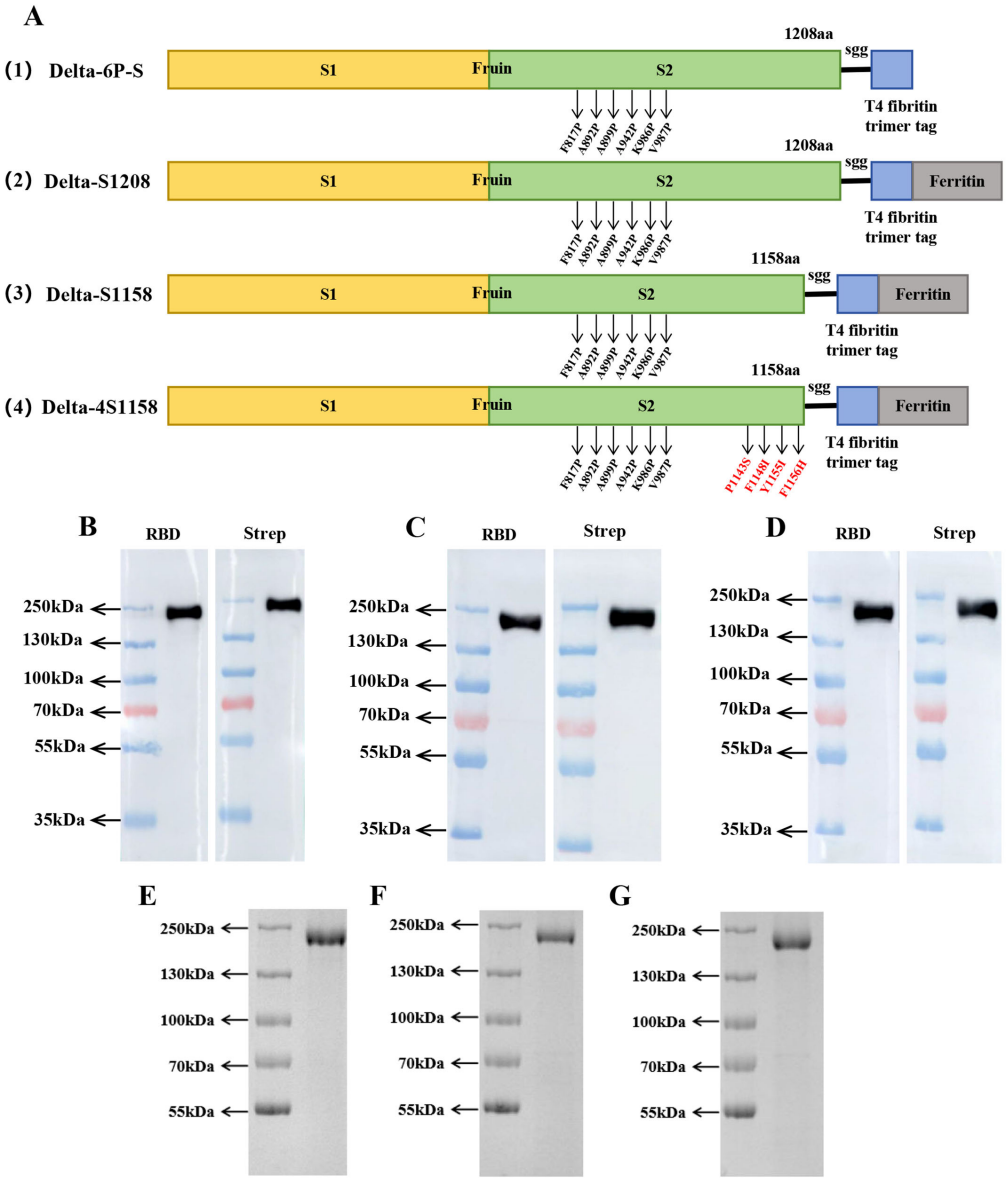
Immunogenic design and purification of ferritin nanoparticles. **(A)** Delta-6P-S recombinant protein, Delta-S1208, Delta-S1158, and Delta-4S1158 nanoparticle design and schematic diagram. **(B–D)** Characterization of Delta-S1208, Delta-S1158, and Delta-4S1158 nanoparticles by Western blot. **(E–G)** SDS-PAGE gel showing Delta-S1208, Delta-S1158, and Delta-4S1158 nanoparticle bands.

**Figure 2:**
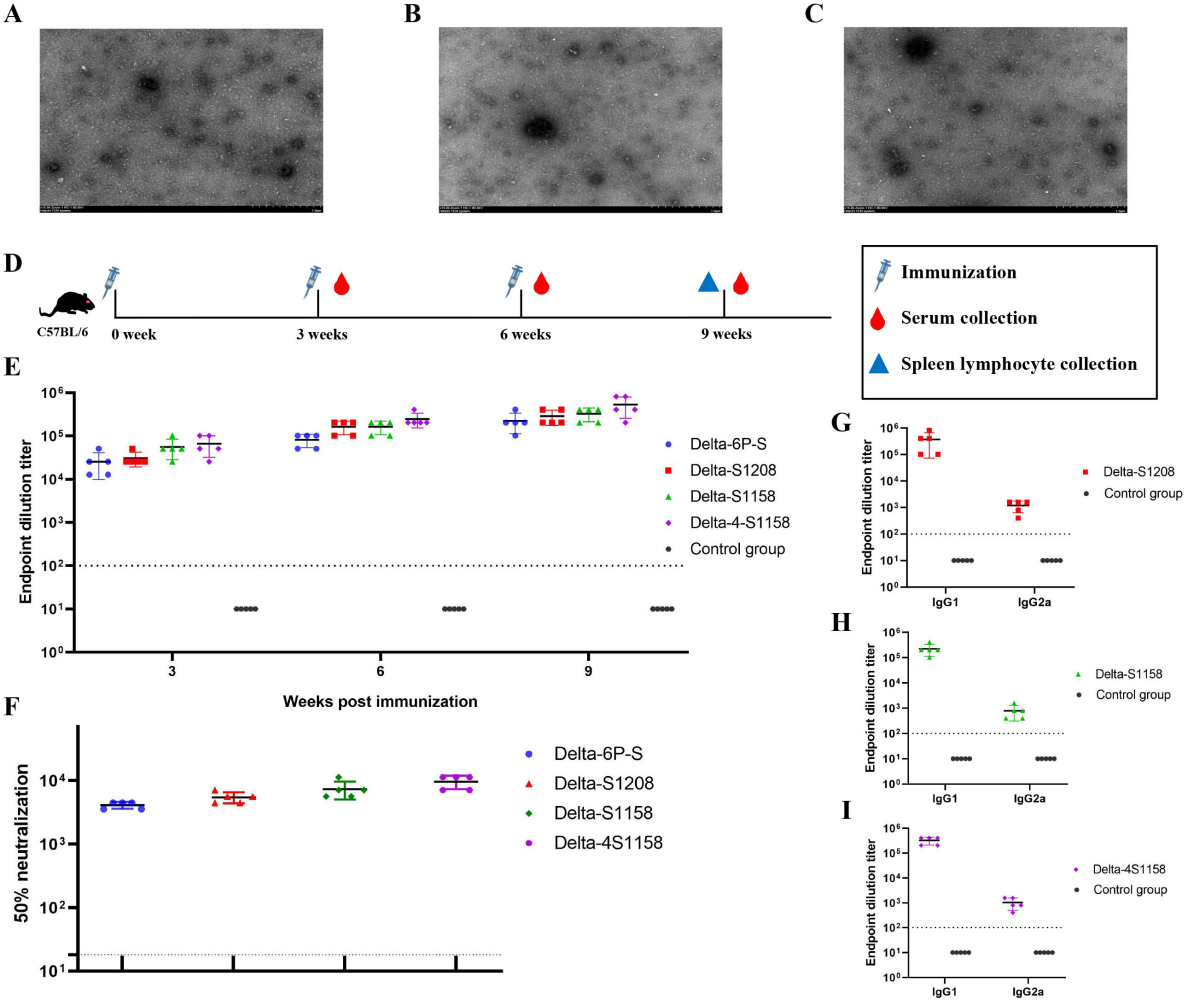
Electron microscopy observation of Delta-S1208, Delta-S1158, and Delta-4S1158 nanoparticles and induction of immune response in C57BL/6J mice. **(A–C)** Negative staining electron microscopy images of Delta-S1208, Delta-S1158, and Delta-4S1158 nanoparticles. Scale bar = 1 μm. **(D)** Immunization and evaluation procedures of Delta-S1208, Delta-S1158, and Delta-4S1158 nanoparticles in C57BL/6J mice. **(E)** The S-specific IgG antibodies of Delta-6P-S recombinant protein, Delta-S1208, Delta-S1158, and Delta-4S1158 nanoparticles in serum at 3, 6, and 9 weeks post-immunization (*n* = 5). **(F)** The 50% neutralization titers of Delta-6P-S recombinant protein, Delta-S1208, Delta-S1158, and Delta-4S1158 nanoparticle groups against the Delta variant (*n*=5). **(G–I)** Antibody subclass detection of Delta-S1208, Delta-S1158, and Delta-4S1158 nanoparticle groups. Individual animal values are indicated by colored symbols. Source data are provided as a Source Data file, and the data are presented as mean values ± SEM. The horizontal dashed line indicates the lower limit of detection (LLOD).

Based on the structure of the previously developed “Delta-6P-S” candidate vaccine (stabilized in the pre-fusion conformation), we designed four immunogens for evaluation: (1) “Delta-6P-S” (reference candidate); (2) Delta-S1208: the original design linked to *H. pylori* ferritin; (3) Delta-S1158: derived from Delta-S1208 by truncating the S protein, removing the helix region, while retaining the T4 fibritin trimer tag and six proline mutations; and (4) Delta-4S1158: derived from Delta-S1158 with four additional amino acid substitutions (P1143S, F1148I, Y1155I, and F1156H), as described by Joyce et al. (2021) (**Figure 1A**). The sequences were codon-optimized, cloned into the pSN vector, transfected into CHO-S, and purified by Strep-Tactin^®^XT affinity chromatography. Western blot analysis with RBD-pAb and Strep-tag mAb confirmed protein expression. The purified ferritin nanoparticles migrated at 180 kDa (**Figures 1B–D**). Sodium dodecyl sulfate polyacrylamide gel electrophoresis (SDS-PAGE) showed a single band (**Figures 1E–G**). Transmission electron microscopy revealed nanoparticles with uniform size and morphology (**Figures 2A–C**).

### 3.2 Immune responses induced by differently designed ferritin nanoparticles in C57BL/6J mice

**Figure 3:**
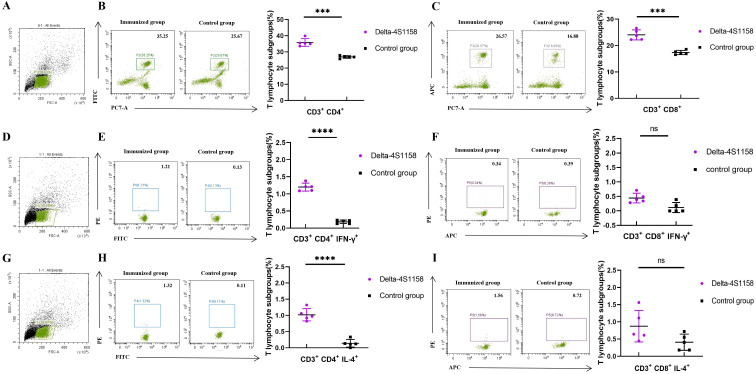
Flow cytometry detection of mouse spleen lymphocytes in the Delta-4S1158 nanoparticle group. Representative plots from each group are shown. **(A, D, G)** Gating strategy of flow cytometry. **(B, C)** Delta-4S1158 nanoparticles induced T lymphocyte differentiation in C57BL/6J mice. **(E, F, H, I)** Delta-4S1158 nanoparticles induced intracellular cytokines in T lymphocytes of C57BL/6J mice. Individual animal values are indicated by colored symbols, and the data are presented as mean values ± SEM.

To compare the immunogenicity of structurally distinct nanoparticles, C57BL/6J mice (5 groups, *n*=5) were immunized at 3-week intervals. Serum S-specific IgG titers were measured (**Figure 2D**). High antibody levels were detected after the first dose, with geometric mean titers (GMTs) ranging from 1:25,600 to 1:66,560. After the third booster, GMTs were 1:225,280 (Delta-6P-S), 1:286,720 (Delta-S1208), 1:327,680 (Delta-S1158), and 1:532,480 (Delta-4S1158) (**Figure 2E**). The control group exhibited background levels of the signal (**Figure 2E**). Neutralizing antibody titers against the Delta variant followed a similar trend. The Delta-4S1158 group reached an NT50 of 1:9,757, which was 2.34-fold higher than Delta-6P-S, 1.76-fold higher than Delta-S1208, and 1.31-fold higher than Delta-S1158 (**Figure 2F**). Antibody subclasses were predominantly IgG1, consistent with a Th2-biased response (**Figures 2G–I**). Thus, immunization with Delta-4S1158 nanoparticles induced a higher level of immune response in C57BL/6J mice. The spleen lymphocytes were detected by flow cytometry. Flow cytometry showed significantly increased frequencies of CD3^+^CD4^+^ and CD3^+^CD8^+^ T cells compared with controls (*p*<0.05; **Figures 3B, C**). Intracellular cytokine staining revealed higher proportions of CD3^+^CD4^+^IFN-γ^+^ and CD3^+^CD4^+^IL-4^+^ T cells in immunized mice (*p*<0.05), indicating enhanced Th2-type responses (**Figures 3E, F, H, I**). A preliminary *in vivo* safety assessment showed no apparent adverse effects in immunized mice (**Supplementary Figure S1**).

### 3.3 Design, expression, purification, and immune response induced in C57BL/6J mice by JN.1-4S1158

**Figure 4:**
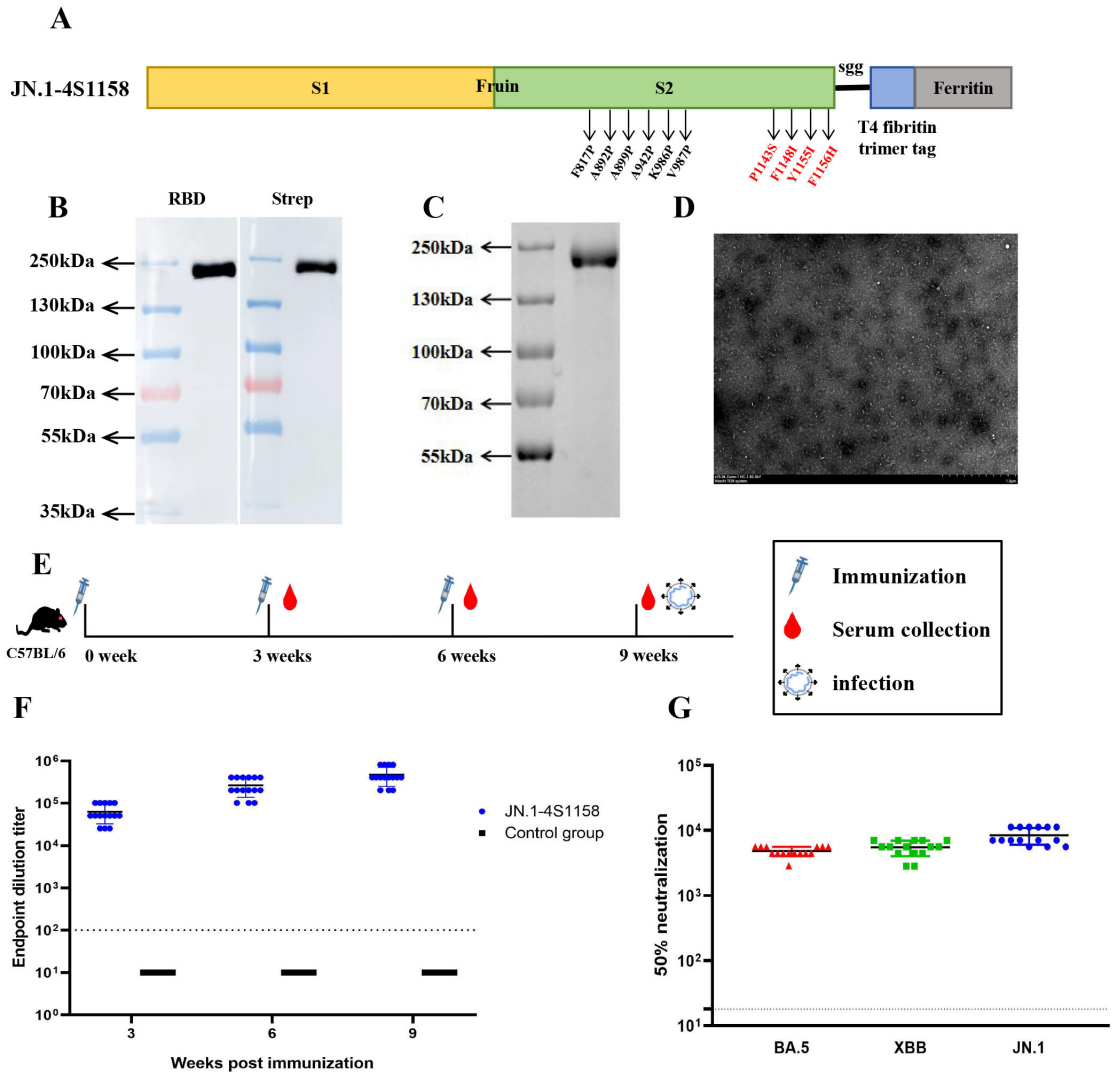
Immunogen design and purification of JN.1-4S1158 nanoparticles and induced immune response in C57BL/6J mice. **(A)** JN.1-4S1158 nanoparticle design and schematic diagram: six proline mutations and four additional mutation sites. **(B)** Characterization of JN.1-4S1158 nanoparticles by Western blot. **(C)** SDS-PAGE gel showing the JN.1-4S1158 nanoparticle band. **(D)** Negative staining electron microscopy images of JN.1-4S1158 nanoparticles. Scale bar = 1 μm. **(E)** Immunization and evaluation procedures of JN.1-4S1158 nanoparticles in C57BL/6J mice. **(F)** The S-specific IgG antibodies of JN.1-4S1158 nanoparticles in serum at 3, 6, and 9 weeks post-immunization (*n* = 15). **(G)** The 50% neutralization titers of JN.1-4S1158 nanoparticles against different Omicron variants (*n* = 15). Individual animal values are indicated by colored symbols, and the data are presented as mean values ± SEM. Source data are provided as a Source Data file. The horizontal dashed line indicates the lower limit of detection (LLOD).

To address emerging Omicron variants, we developed JN.1-4S1158, based on the Delta-4S1158 design but incorporating the Omicron JN.1 S protein sequence with six proline substitutions and the four additional stabilizing mutations (P1143S, F1148I, Y1155I, and F1156H) (**Figure 4A**). Expression in CHO-S cells and purification were confirmed by Western blot, which showed a band at ~180 kDa (**Figure 4B**). SDS-PAGE revealed a single band (**Figure 4C**), and electron microscopy confirmed nanoparticle formation (**Figure 4D**). Mice (2 groups, *n*=15) were immunized on a prime-boost regimen. On week 3, the GMT of S-specific IgG was 1:63,146, rising to 1:477,867 by week 9 (**Figure 4F**). The control group showed background levels. Neutralization assays demonstrated cross-protection: NT50s were 1:4,811 (BA.5), 1:5,493 (XBB), and 1:8,449 (JN.1) (**Figure 4G**).

### 3.4 JN.1-4S1158 protection against Omicron variants in C57BL/6J mice

**Figure 5:**
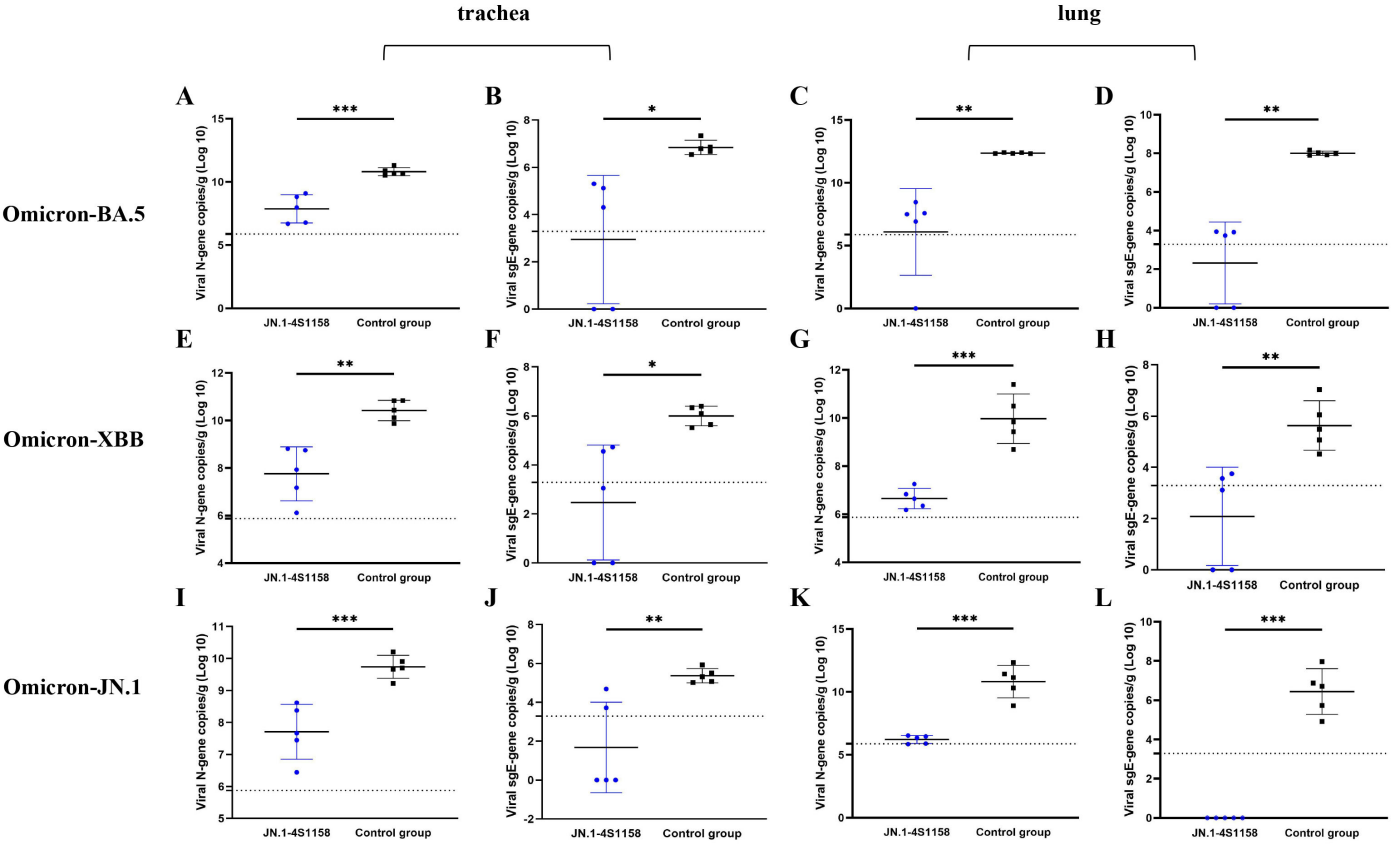
Viral N gene and sgE gene copies of trachea and lungs in C57BL/6J mice at 4 dpi. **(A, C, E, G, I, K)** Comparison of viral gene copy measurements of qPCR targeting viral N gene from tracheas **(A, E, I)** and lungs **(C, G, K)** between the immunized groups and the control groups after challenge with Omicron BA.5, XBB, and JN.1 variants at 4 dpi. **(B, D, F, H, J, L)** Comparison of viral gene copy measurements of qPCR targeting viral N gene from tracheas **(B, F, J)** and lungs **(D, H, L)** between the immunized groups and the control groups after challenge with Omicron BA.5, XBB, and JN.1 variants at 4 dpi. Data of the JN.1-4S1158 nanoparticle groups are shown in blue. Data of the control groups are shown in black. Individual animal values are indicated by colored symbols. Comparisons were performed by multiple *t*-test; *p ≤ 0.05, **p ≤ 0.01, ***p ≤ 0.001. Source data are provided as a Source Data file. The horizontal dashed line indicates the lower limit of detection (LLOD).

**Figure 6:**
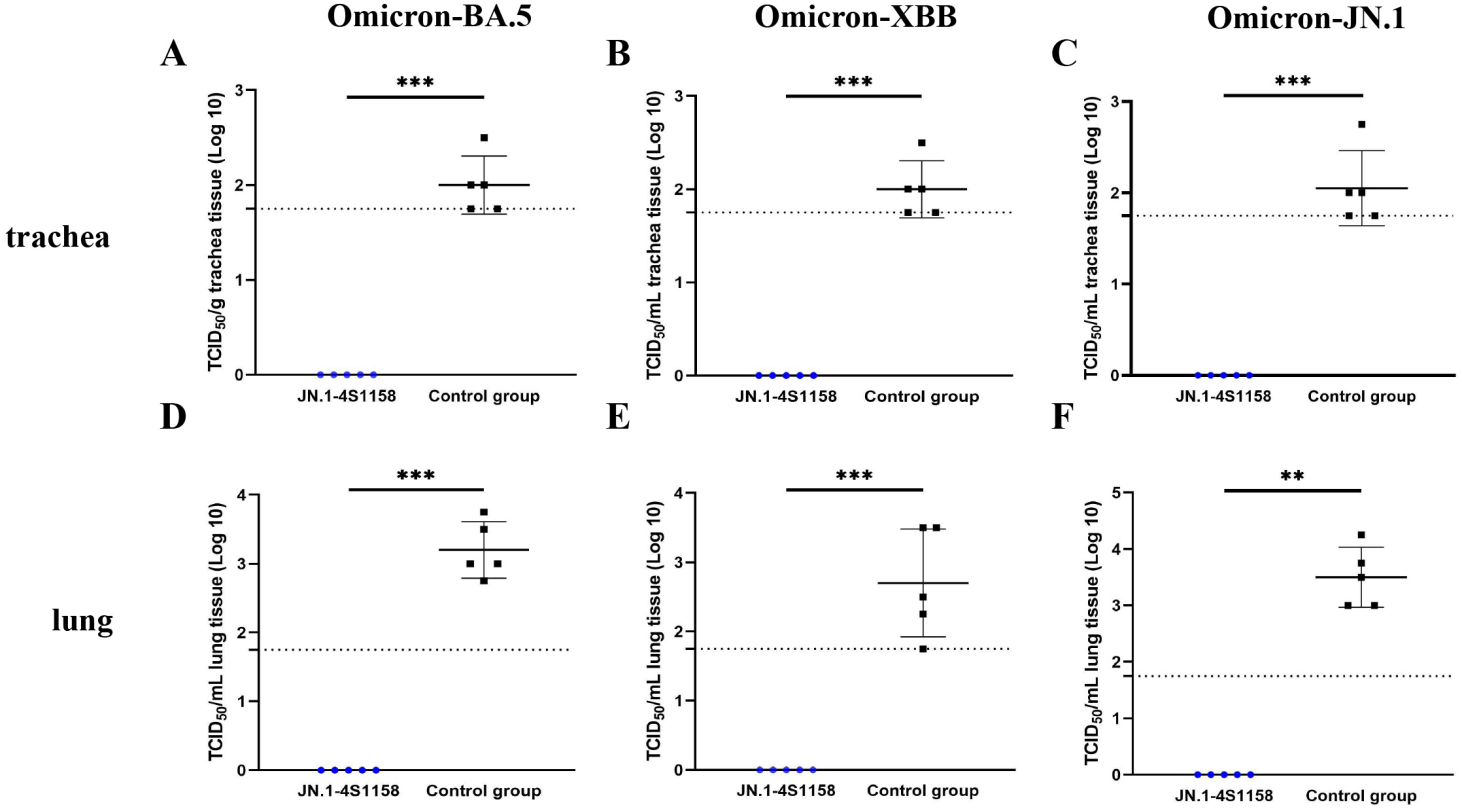
Protection of JN.1-4S1158 nanoparticles against Omicron variant infection in C57BL/6J mice. **(A–F)** Viral load in trachea **(A–C)** and lung **(D–F)** homogenates at 4 days post-Omicron BA.5, XBB, and JN.1 variant challenge. Bars represent the mean viral load (*n* = 5). Data of JN.1-4S1158 nanoparticle groups are presented in blue. Data from the control groups are presented in black. Individual animal values are indicated by colored symbols. Comparisons were performed by multiple t-test; *p ≤ 0.05, **p ≤ 0.01, ***p ≤ 0.001. Source data are provided as a Source Data file. The horizontal dashed line indicates the lower limit of detection (LLOD).

**Figure 7:**
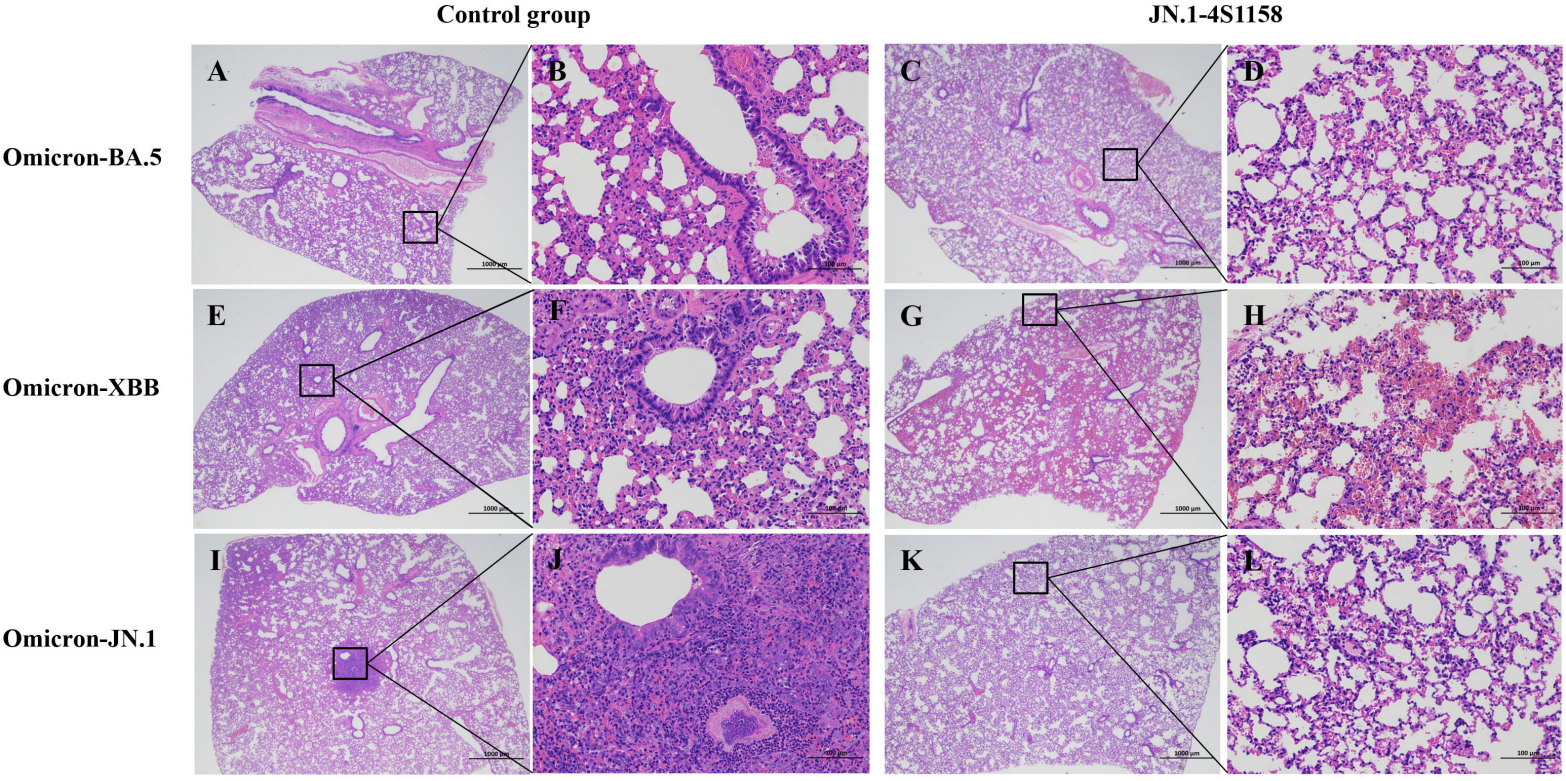
Pathological observation of lungs in C57BL/6J mice challenged with Omicron variants. Representative plots from each group are shown. **(A, E, I)** Lungs of C57BL/6J mice from control groups at 4 dpi. **(A)** Moderate alveolar wall thickening and inflammatory cell infiltration; scale bar = 1,000 μm. **(E)** Moderate alveolar wall thickening and inflammatory cell infiltration with bleeding; scale bar = 1,000 μm. **(I)** Moderate alveolar wall thickening and inflammatory cell infiltration with bleeding; scale bar = 1,000 μm. **(B, F, J)** Scale bar = 100 μm. **(C, G, K)** Lungs of C57BL/6J mice from JN.1-4S1158 nanoparticle groups at 4 dpi. **(C)** Mild alveolar wall thickening; scale bar = 1,000 μm. **(G)** Mild inflammatory cell infiltration with bleeding; scale bar = 1,000 μm. **(K)** Mild inflammatory cell infiltration; scale bar = 1,000 μm **(D, H, J)**.

To evaluate protection, immunized and control mice (*n* = 5 per group) were challenged intranasally with Omicron BA.5, XBB, or JN.1 (100 μL, 10^4.75^ TCID_50_/mL). In JN.1-challenged mice, lung viral RNA levels were 10^6.22^ copies/g compared with 10^10.82^ in controls (*p*<0.05; **Figures 5K, L**). Tracheal viral loads were 10^7.71^ vs. 10^9.74^ copies/g, respectively (*p*<0.05; **Figures 5I, J**). Infectious virus was undetectable in the lungs and trachea of immunized mice but present at high levels in controls (**Figures 6C, F**). Similar protection was observed against BA.5 and XBB (**Figures 5A–H**, **6A–D**). Histopathological analysis revealed that control mice exhibited hemorrhage, inflammatory infiltration, and alveolar wall thickening, whereas vaccinated mice showed minimal to no lung pathology (**Figure 7**). In summary, JN.1-4S1158 nanoparticles induced robust antibody and T-cell responses and protected against multiple Omicron variants in C577BL/6J mice.

## 4 Discussion

The continued evolution of SARS-CoV-2 presents a significant challenge to global public health (Li et al., 2025). Large numbers of people remain infected with emerging Omicron variants (Giombini et al., 2025; Naveed Siddiqui et al., 2025). Currently, several vaccines have been developed for SARS-CoV-2, which can induce an excellent protective immune response; however, this protective effect is reduced against new variants (Saha and Ghosh Roy, 2025). Ferritin is an attractive vaccine platform because it is safe, stable, and can display antigens on its surface without disrupting the self-assembly of the nanoparticles. This versatility makes ferritin nanoparticles promising candidates for vaccine development and antigen delivery (Yang et al., 2024). While previous studies have demonstrated the strong immunogenicity of ferritin nanoparticle vaccines, relatively few have evaluated their efficacy against newer Omicron variants (Joyce and King, 2022; Wuertz and Barkei, 2021). In previous studies, we confirmed that the six-proline stabilized S-6P construct provided stronger protection than S-2P in mice and golden hamsters against SARS-CoV-2 infection (Feng et al., 2023). Other studies have further demonstrated that truncation of S-2P combined with four amino acid substitutions (P1143S, F1148I, Y1155I, and F1156H) enhances the immune response (Joyce et al., 2021). Building on this knowledge, we compared the immunogenicity of ferritin nanoparticles presenting S proteins with different structural designs in C57BL/6J mice and identified the optimal configuration. This led to the development of the “JN.1-4S1158” nanoparticle vaccine, which elicited strong immune responses and provided effective protection against Omicron BA.5, XBB, and JN.1 variants. No infectious virus was detected in the lungs or trachea of vaccinated animals, and levels of both the N and sgE genes were near the limit of detection, in contrast to the high viral burdens observed in control mice.

Since the receptor binding domain (RBD) of wild-type SARS-CoV-2 does not interact with mouse ACE2, hACE2 transgenic mouse models have commonly been used to test vaccine efficacy (Park et al., 2024; Thiede and Dick, 2024). Mutations such as N501Y expanded the host range by improving binding to mouse ACE2 (Halfmann and Iida, 2022; Wei et al., 2021). In this study, we used C57BL/6J mice to assess immunogenicity and cross-protection. We found that S-specific IgG titers increased significantly following immunization, with nanoparticle formulations eliciting higher antibody levels than monomeric proteins. Neutralizing antibody titers followed the same trend, consistent with the principle that multivalent antigen display enhances immunogenicity in line with prior vaccine studies (Sankhala et al., 2024; Wu et al., 2024). Among the designs tested, Delta-4S1158 nanoparticles induced the strongest immune response, confirming that this truncated S-6P design with four stabilizing mutations is well-suited for ferritin display.

The emergence of the Omicron JN.1 variant has raised concern due to its pronounced immune evasion compared with earlier variants (Kaku et al., 2024; Li et al., 2024). Although there is no clinical evidence that JN.1 causes more severe disease than other variants, mutations such as L455S in the RBD may enhance infectivity and broaden tissue tropism (Chang et al., 2025; Tian et al., 2025; Yang et al., 2024). In our study, the JN.1-4S1158 vaccine elicited high levels of cross-neutralizing antibodies against Omicron BA.5, XBB, and JN.1 variants. While neutralization titers against BA.5 and XBB were lower than those against JN.1, they remained within the range considered protective, consistent with findings from other recent studies (Ao et al., 2025; Yang et al., 2025). Challenge experiments confirmed these results: vaccinated mice showed no detectable infectious virus in the lungs or trachea, in contrast to high viral titers in controls. The ability of JN.1-4S1158 to confer cross-protection suggests that it may be effective against multiple Omicron sublineages, although its activity against other emerging variants requires further study. Concerns about antibody-dependent enhancement (ADE) have been raised in the context of SARS-CoV and MERS-CoV vaccines, where enhanced respiratory disease was reported in animal models (Karthik et al., 2020; Li et al., 2021). However, in preclinical models and large clinical trials of SARS-CoV-2 vaccines, such effects have not been observed (Gao and Bao, 2020; Wang et al., 2020). In our study, preliminary safety assessments revealed no evidence of pathology or adverse effects in vaccinated mice, supporting the biosafety of the ferritin nanoparticle platform.

In summary, the JN.1-4S1158 ferritin nanoparticle vaccine demonstrated strong preclinical efficacy against Omicron BA.5, XBB, and JN.1 variants in mice. These findings highlight the potential of ferritin-based vaccines as adaptable platforms for combating emerging SARS-CoV-2 variants. Further studies, including evaluation in additional animal models and eventual clinical trials, are warranted to validate safety, durability, and breadth of protection.

## Data Availability

The original contributions presented in the study are included in the article/**Supplementary Material**. Further inquiries can be directed to the corresponding authors.
