# Supplementary figures and images for: Ferritin nanoparticle vaccine displaying optimized spike protein confers broad protection against Omicron subvariants
Source: Front Cell Infect Microbiol. 2025 Nov 4;15:1676592. doi: 10.3389/fcimb.2025.1676592 (PMC12623353; doi:10.3389/fcimb.2025.1676592)

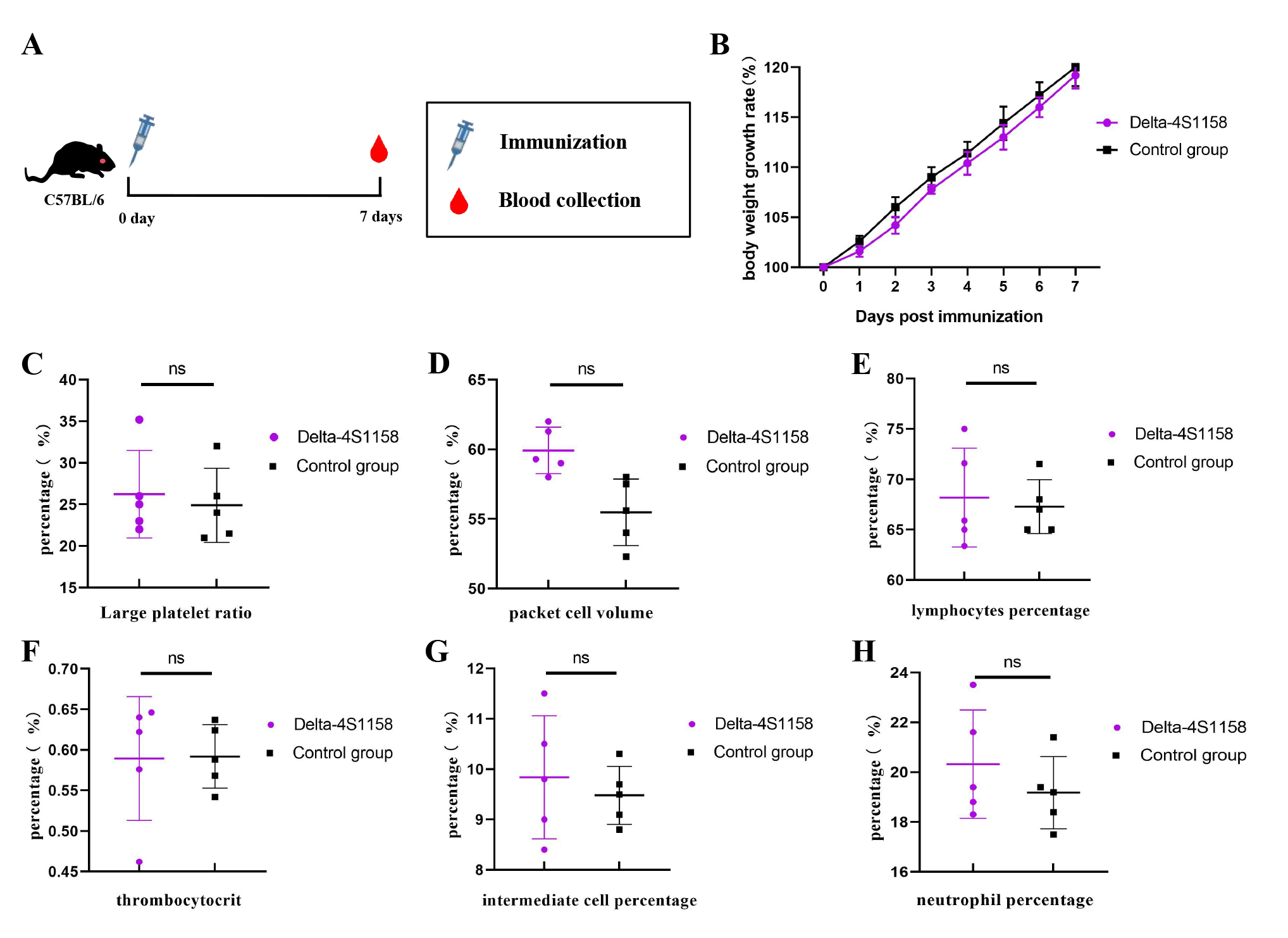

Supplement: Supplementary file 1 [file Image1.jpeg]
